# Preventive and Therapeutic Effects of Krill Oil on Obesity and Obesity-Induced Metabolic Syndromes in High-Fat Diet-Fed Mice

**DOI:** 10.3390/md20080483

**Published:** 2022-07-27

**Authors:** Seung-Min Hwang, Yeong Uk Kim, Jong-Kyu Kim, Yoon-Seok Chun, Young-Sam Kwon, Sae-Kwang Ku, Chang-Hyun Song

**Affiliations:** 1 Department of Veterinary Surgery, College of Veterinary Medicine, Kyungpook National University, Daegu 41566, Korea; vet1st@knu.ac.kr (S.-M.H.); kwon@knu.ac.kr (Y.-S.K.); 2 Department of Urology, College of Medicine, Yeungnam University, Daegu 42415, Korea; jojo9174@hanmail.net; 3 AriBnC Co., Ltd., Yongin 16914, Korea; swrhrnak@gmail.com (J.-K.K.); ceochun@aribnc.com (Y.-S.C.); 4 Department of Anatomy and Histology, College of Korean Medicine, Daegu Haany University, Gyeongsan 38610, Korea

**Keywords:** obesity, diabetes, NAFLD, T2D, dyslipidemia, HFD, steatosis, metformin, marine, PUFA

## Abstract

Obesity increases the risks of metabolic syndromes including nonalcoholic fatty liver disease (NAFLD), diabetic dyslipidemia, and chronic kidney disease. Dietary krill oil (KO) has shown antioxidant and anti-inflammatory properties, thereby being a therapeutic potential for obesity-induced metabolic syndromes. Thus, the effects of KO on lipid metabolic alteration were examined in a high-fat diet (HFD)-fed mice model. The HFD model (*n* = 10 per group) received an oral gavage with distilled water as a control, metformin at 250 mg/kg, and KO at 400, 200, and 100 mg/kg for 12 weeks. The HFD-induced weight gain and fat deposition were significantly reduced in the KO treatments compared with the control. Blood levels were lower in parameters for NAFLD (e.g., alanine aminotransferase, and triglyceride), type 2 diabetes (e.g., glucose and insulin), and renal dysfunction (e.g., blood urea nitrogen and creatinine) by the KO treatments. The KO inhibited lipid synthesis through the modification of gene expressions in the liver and adipose tissues and adipokine-mediated pathways. Furthermore, KO showed hepatic antioxidant activities and glucose lowering effects. Histopathological analyses revealed that the KO ameliorated the hepatic steatosis, pancreatic endocrine/exocrine alteration, adipose tissue hypertrophy, and renal steatosis. These analyses suggest that KO may be promising for inhibiting obesity and metabolic syndromes.

## 1. Introduction

Obesity is a common metabolic disorder caused by high-caloric energy intake and reduced energy expenditure over time [1]. The prevalence of obesity has increased; obese individuals comprised approximately 13% of adults worldwide in 2016, and obesity is estimated to affect one in five adults by 2025 if these trends continue [2,3]. Adipose tissue, particularly white adipose tissue, not only provides lipid storage as an energy reservoir, but also act as an integral part of the endocrine function releasing various adipokines for lipid metabolism [4]. However, excessive high-fat intake overwhelming the storage capacity of the adipose tissue induces endocrine dysfunction and then body fat disposition, especially in the abdominal region, followed by lipotoxic metabolic stress [5]. From this progresses chronic low-grade inflammation and oxidative stress, resulting in metabolic alterations in the pancreas, liver, and skeletal muscle. The metabolic alterations cooperatively promote insulin resistance and hyperglycemia, which increases the risk of the relevant metabolic syndromes such as nonalcoholic fatty liver disease (NAFLD), type 2 diabetes (T2D), dyslipidemia, and chronic kidney disease [6]. The occurrence of metabolic syndromes is currently increasing along with the exponential rise in obesity, representing that obesity is a major public health challenge [7]. Thus, there is an unmet need of comprehensive therapeutic strategies targeting obesity and obesity-induced metabolic syndromes.

Primary prevention and treatments of obesity is dietary and life-style modification: moderate exercise and dietary changes through reduced total fat intake and intakes of less saturated fat and more unsaturated fatty acids could lower weight gains and the incidence of metabolic syndromes [8]. The treatment guidelines recommend certain pharmacological drugs in NAFLD patients with T2D or nonalcoholic steatohepatitis; however, there are no effective and safe drugs targeting both NAFLD and T2D [9]. Metformin, as the AMP-activated protein kinase (AMPK) activator, is the most widely used for overweight patients with T2D, and it also has favorable effects on NAFLD by reducing insulin resistance and hepatic triglyceride accumulation [10]. It is reported that metformin prevents weight gain and the steatosis by elevating GDF15, a peptide hormone that responds to stressors and lowers glucose levels in the absence of GDF15 action [11]. However, the efficacy of metformin is limited in improving the histopathological changes of NAFLD and is controversial [12]. Furthermore, its clinical use has risks of increasing lactic acidosis in comorbid diseases with liver or kidney dysfunction [13]. Peroxisome proliferator-activated receptor (PPAR)γ agonist is effective against NAFLD with and without T2D; however, it has a disadvantageous safety profile such as overweight and congestive heart failure [14]. In addition to chemical drugs, diet interventions of natural bioactive compounds (e.g., polyphenols and certain fatty acids) possessing antioxidant and anti-inflammatory properties have emerged as promising therapeutic sources for metabolic syndromes [15,16]. Although numerous products are available for treating obesity and metabolic syndromes, candidate drugs have insufficient evidence to support their clinical efficacy and safety [17].

Recent studies have reported that various compositions of ingested fatty acids (FAs) differentially affect lipid metabolism and regulation of inflammatory responses. Dietary saturated FAs promote hepatic and visceral fat deposition, leading to metabolic alteration and inflammation, while polyunsaturated FAs (PUFAs) of omega (n)-3 groups inhibit obesity and metabolic alterations involving insulin resistance, dyslipidemia, and inflammation [18,19,20]. Dietary n-3 PUFAs, especially long-chain n-3 PUFAs, including docosahexaenoic acid (DHA) and eicosapentaenoic acid (EPA), have anti-inflammatory and antioxidant properties, which improve insulin resistance and lipid metabolism and ameliorate obesity and dyslipidemia [21,22]. Major sources of long-chain n-3 PUFAs originate from fish and other marine organisms [23].

Krill oil (KO), extracted from Antarctic krill (*Euphausia superba*), is a rich alternative source of n-3 PUFAs containing a high proportion (30–65%) of DHA and EPA in the form of phospholipids (mainly phosphatidylcholine) [22,24]. Given that EPA and DHA bound to phospholipids in KO possess higher bioavailability and absorption rates compared with the triglycerides bound to n-3 PUFAs in fish oil, KO may have more potential in treating metabolic syndromes [24]. KO also contains antioxidant astaxanthin and vitamins A and E [25,26]. Many studies have shown that dietary or supplementary KO inhibits weight gain in a high-fat diet (HFD)-induced obese animal model and ameliorates the metabolic syndromes of NAFLD, T2D, and dyslipidemia by reducing lipid contents and insulin resistance (reviewed in [24,27,28,29,30,31,32,33,34,35,36]). In addition, dietary EPA and DHA from KO have shown inhibitory effects on hepatic steatosis and the gene expressions involving glucose production and lipid synthesis but nonsignificant or inconsistent results in blood levels of lipid and glucose/insulin [37,38,39]. On the other hand, there have been a few studies regarding the direct effects of KO via an oral gavage [40,41,42] and no reports supporting histopathological improvements of metabolic alterations in the multiorgans [40,41,42]. Clinical application has proven the antidyslipidemic effects of KO by reducing blood triglycerides and cholesterol in patients with hyperlipidemia [43] and obese subjects [44]; however, it there have been inconsistent results in blood glucose levels [43,45]. The application of EPA/DHA has not been reported yet in patients. Thus, comprehensive biochemical and histopathological analyses were conducted to examine the effects of KO on lipid metabolic alteration in multiorgans, including the liver, pancreas, adipose tissues, and kidney, after an oral gavage of KO for 12 weeks in a HFD-fed mice model.

## 2. Results

### 2.1. Inhibition of Body Weight Gain

**Figure 1:**
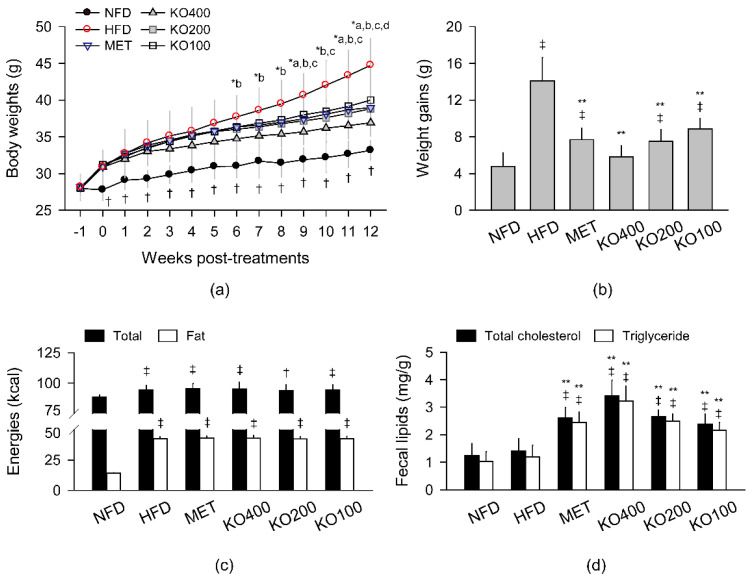
Body weight changes and energy metabolism: (**a**) kinetic weight changes; (**b**) total weight gains for 12 weeks; (**c**) daily intakes of diet energy; (**d**) fecal lipid excretions. Values are expressed as the means ± standard deviations (SDs). ^‡^ *p* < 0.01 and ^†^ *p* < 0.05 versus the NFD. ^*a^, ^*b^, ^*c^, and ^*d^: *p* < 0.05 versus the HFD control (HFD) in the MET, KO400, KO200, and KO100, respectively. ** *p* < 0.01 versus the HFD.

The body weight gain increased in the HFD model by supplying HFD from a week prior to the treatments compared with the normal-fat diet-fed group (NFD), administered with distilled water (DW) as a vehicle (Figure 1). The weight gain was evident in the HFD control with DW compared with the NFD; however, they were reduced in the treatments of metformin at 250 mg/kg (MET) and KO at 400, 200, and 100 mg/kg (KO400, KO200, and KO100, respectively). The kinetic weight changes were examined by two-way analysis of variance (ANOVA) with main factors for the groups and the weeks measuring the weight (Figure 1a). There were significant main effects for the groups (*p* < 0.01) and the weeks (*p* < 0.01). There were also significant interactions between the groups and weeks, meaning the week-dependent weight differences among the groups (*p* < 0.01). Post hoc tests revealed significant increases in the body weight of all HFD model groups for 12 weeks compared with those of the NFD (*p* < 0.05). However, when compared with the HFD control, the weight gains were significantly reduced in the MET in 9, 11, and 12 weeks post-treatments (wpt); in the KO400 in 6–12 wpt; in the KO200 in 9–12 wpt; in the KO100 in 12 wpt (*p* < 0.05). The total weight gain at 12 weeks increased in the HFD control compared with the NFD; however, it was reduced in the treatment groups of MET and KO compared with the HFD control (*p* < 0.01, Figure 1b). The weight gain was not significantly different between the NFD and KO400 groups.

### 2.2. Stimulation of Lipid Excretion

Daily intakes of total food and fat calories were measured every week (Figure 1c). Both calories significantly increased in the HFD model groups regardless of any treatments compared with the NFD (*p* < 0.05), and they were not different in the treatment groups of MET and KO compared with the HFD control. The fecal contents of total cholesterol and triglyceride were measured after all treatments (Figure 1d). The lipid contents were not different between the NFD and HFD control groups; however, they were significantly increased in the MET and KO groups compared with the NFD and HFD control (*p* < 0.01).

### 2.3. Inhibition of Body Fat Deposition

**Figure 2:**
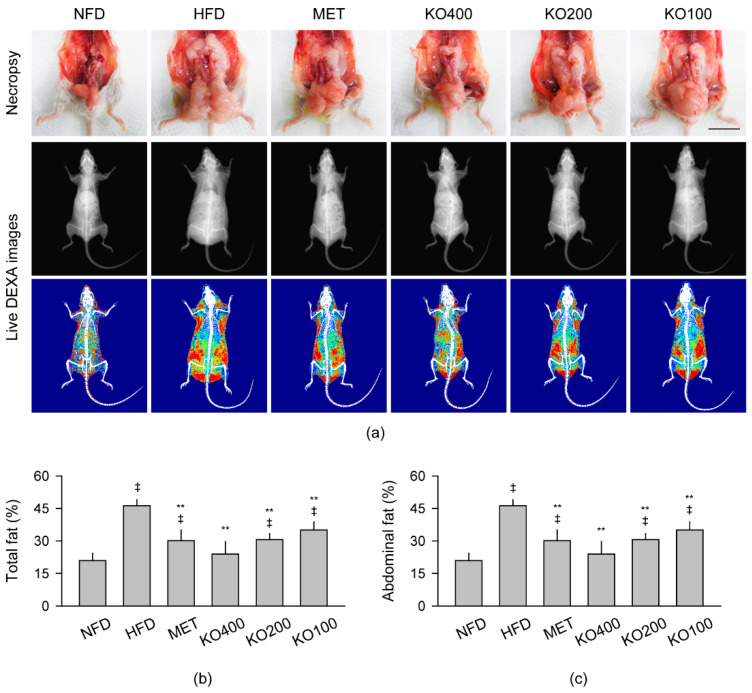
Body fat deposition: (**a**) Representative images of body fat deposition in necropsy and live dual-energy X-ray absorptiometry (DEXA). In the live DEXA images, red, yellow, and blue indicate high-, intermediate-, and low-density fats, respectively. Scale bars = 2 cm. (**b**,**c**) Fat densities in total body and the abdominal region. Values are expressed as the mean ± SD. ^‡^ *p* < 0.01 versus the NFD. ** *p* < 0.01 versus the HFD.

Body fat deposition, especially in the abdominal region, were evident in necropsy of the HFD control; however, it was attenuated in the treatment groups of MET and KO (Figure 2a). The live dual-energy X-ray absorptiometry (DEXA) images were analyzed for the total and abdominal fat deposition. One-way ANOVA showed significant differences in the fat depositions among the groups (*p* < 0.01, Figure 2b,c). The total and abdominal fat deposition was significantly increased in the HFD control compared with the NFD; however, it was reduced in the treatment groups compared with the HFD control (*p* < 0.01). Both fat depositions were not different between the NFD and KO400 groups.

### 2.4. Improvements in Organ Weights Involved in Metabolic Alteration

One-way ANOVA showed significant differences in the absolute organ weights of the liver, kidney, and abdominal/periovarian fat masses and the relative organ weights to the body weight of the pancreas and abdominal/periovarian fat masses among the groups (*p* < 0.01, Table 1). The absolute weights of the liver, kidney, and abdominal/periovarian fat masses increased in the HFD control compared with the NFD (*p* < 0.01); however, they were significantly reduced in the treatment groups of MET and KO compared with the HFD control (*p* < 0.01). The relative weights showed significant increases in the abdominal/periovarian fat masses and decreases in the pancreas in the HFD control compared with the NFD (*p* < 0.01); however, they were significantly reversed in the treatment groups compared with the HFD control (*p* < 0.05).

### 2.5. Improvements in Blood Biochemical Parameters Involved in Metabolic Alteration

The blood biochemical analysis was examined for the severity of the metabolic syndromes as follows (Table 2): for NAFLD and dyslipidemia, alanine aminotransferase (ALT), aspartate aminotransferase (AST), alkaline phosphatase (ALP), lactate dehydrogenase (LDH), gamma-glutamyltransferase (GGT), triglyceride, total cholesterol, and low-/high-density lipoprotein (LDL/HDL) cholesterol; for T2D, glucose, insulin, and blood glycated hemoglobin (HbA1c); for renal dysfunction, blood urea nitrogen (BUN) and creatinine. One-way ANOVA showed significant differences in all of the parameters among the groups (*p* < 0.01). The serum level of HDL cholesterol was reduced in the HFD control compared with the NFD (*p* < 0.01); however, it increased in the treatment groups of MET and KO compared with the HFD control (*p* < 0.01). Conversely, the other levels increased in the HFD control compared with the NFD (*p* < 0.01); however, they were reduced in the treatment groups compared with the HFD control (*p* < 0.05). The levels of HbA1c, glucose, and HDL cholesterol were similar between the NFD and KO400 groups.

### 2.6. Enhancement of Hepatic Antioxidant and Glucose-Regulating Enzyme Activities

**Figure 3:**
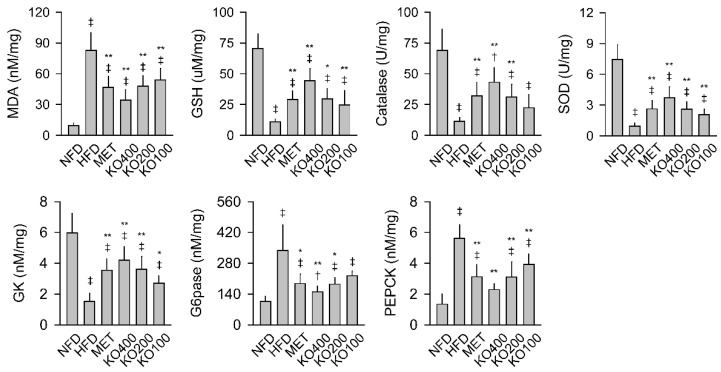
Hepatic antioxidant and glucose-regulating enzyme activities. The levels of hepatic malondialdehyde (MDA) and glutathione (GSH) and the activities of catalase and superoxide dismutase (SOD) were assessed for antioxidant activities, and glucokinase (GK), glucose-6-phosphatase (G6pase), and phosphoenolpyruvate carboxykinase (PEPCK) were assessed for glucose-regulating enzyme activities. Values are expressed as the mean ± SD. ^‡^ *p* < 0.01 and ^†^ *p* < 0.05 versus the NFD. ** *p* < 0.01 and * *p* < 0.05 versus the HFD.

For the hepatic antioxidant effects of KO, the tissue levels of malondialdehyde (MDA) for the lipid peroxidation, contents of glutathione (GSH) as an endogenous antioxidant, and the activities of antioxidant enzymes of catalase and superoxide dismutase (SOD) were assessed (Figure 3). There were significant differences in all of the antioxidant parameters among the groups (*p* < 0.01). In the HFD control compared with the NFD, the levels of MDA increased, while the contents of GSH and activities of catalase and SOD were reduced (*p* < 0.01). However, all levels were significantly reversed in the treatment groups of MET and KO compared with the HFD control (*p* < 0.05). In addition, there were significant differences in the activities of the hepatic glucose-regulating hepatic enzymes of glucokinase (GK), glucose-6-phosphatase (G6pase), and phosphoenolpyruvate carboxykinase (PEPCK) among the groups (*p* < 0.01). The GK activity was reduced in the HFD control compared with the NFD, while the G6pase and PEPCK activities increased (*p* < 0.01). However, the activities were significantly reversed in the treatment groups compared with the HFD control (*p* < 0.05). The PEPCK activities were similar between the NFD and KO400 groups.

### 2.7. Modification of Gene Expressions Involved in Metabolic Alteration

**Figure 4:**
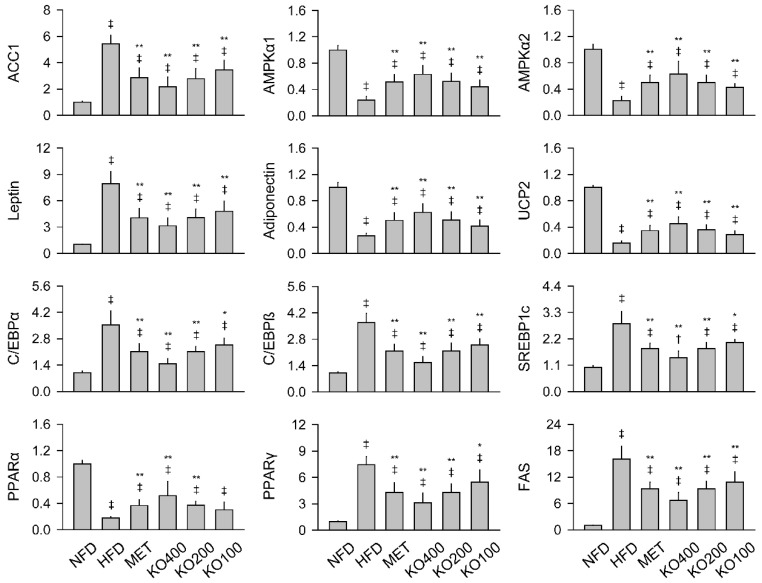
Gene expressions involved in metabolic alteration. Gene expressions of acetyl-CoA carboxylase 1 (ACC1), AMP-activated protein kinase (AMPK)α1, and AMPKα2 were assessed in the liver, and the expressions of leptin, adiponectin, uncoupling protein (UCP)2, CCAAT-enhancer-binding protein (C/EBP)α, C/EBPβ, sterol-regulatory-element-binding protein 1c (SREBP1c), peroxisome proliferator-activated receptor (PPAR)α, PPARγ, and fatty acid synthase (FAS) were assessed in the periovarian fat tissues. Values are expressed as the mean ± SD. ^‡^ *p* < 0.01 and ^†^ *p* < 0.05 versus the NFD. ** *p* < 0.01 and * *p* < 0.05 versus the HFD.

There were significant differences in the hepatic mRNA expressions for acetyl-CoA carboxylase 1 (ACC1), an enzyme that catalyzes carboxylation of acetyl-CoA to malonyl-CoA for fatty acid synthesis, AMPK-α1 and -α2, as master regulators of cellular energy homeostasis, among the groups (*p* < 0.01, Figure 4). In the HFD control compared with the NFD, the ACC1 was upregulated, while the AMPK-α1 and -α2 were downregulated (*p* < 0.01). However, compared to the HFD control, the treatments of MET and KO downregulated ACC1 and upregulated AMPK-α1 and -α2 (*p* < 0.01). Further gene expressions for PPAR-α and -γ, uncoupling protein (UCP)2, CCAAT-enhancer-binding protein (C/EBP)α, C/EBPβ, fatty acid synthase (FAS), sterol-regulatory-element-binding protein 1c (SREBP1c), and adipokines of leptin and adiponectin were assessed in the periovarian fat tissue as a predominant region of white adipose tissues. The expressions were also significantly different among the groups (*p* < 0.01). The C/EBPα, C/EBPβ, SREBP1c, PPARγ, FAS, and leptin were upregulated in the HFD control compared with the NFD, while the PPARα, UCP2, and adiponectin were downregulated (*p* < 0.01). However, the lipid synthesis-related gene expressions were significantly reversed in the treatment groups of MET and KO, except for the PPARα in the KO100 compared with the HFD control (*p* < 0.05).

### 2.8. Improvements in Histopathological Changes Involved in Metabolic Alteration

#### 2.8.1. Histopathological Improvements in the Liver

**Figure 5:**
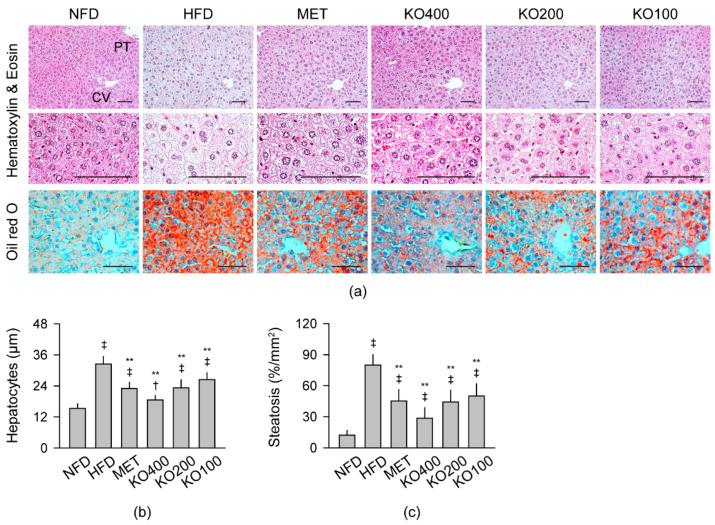
Histopathological changes in the liver: (**a**) Representative images in stains with hematoxylin and eosin and Oil red O. CV = central vein; PT = portal triad. Scale bars = 50 μm. (**b**,**c**) Sizes of the hepatocytes and Oil red O-stained areas. Values are expressed as the mean ± SD. ^‡^ *p* < 0.01 and ^†^ *p* < 0.05 versus the NFD. ** *p* < 0.01 versus the HFD.

The hepatic hypertrophy and steatosis were evident in hematoxylin and eosin (HE) and Oil red O stains in the HFD control; however, they were mild in the treatment groups of MET and KO (Figure 5). Histomorphometric analyses showed significant differences in the hepatocyte sizes and oil red O-stained regions among the groups (*p* < 0.01). The hepatocyte sizes and the stained regions increased in the HFD control compared with the NFD (*p* < 0.05); however, they were significantly reduced in the treatment groups compared with the HFD control (*p* < 0.01).

#### 2.8.2. Histopathological Improvements in the Pancreas

**Figure 6:**
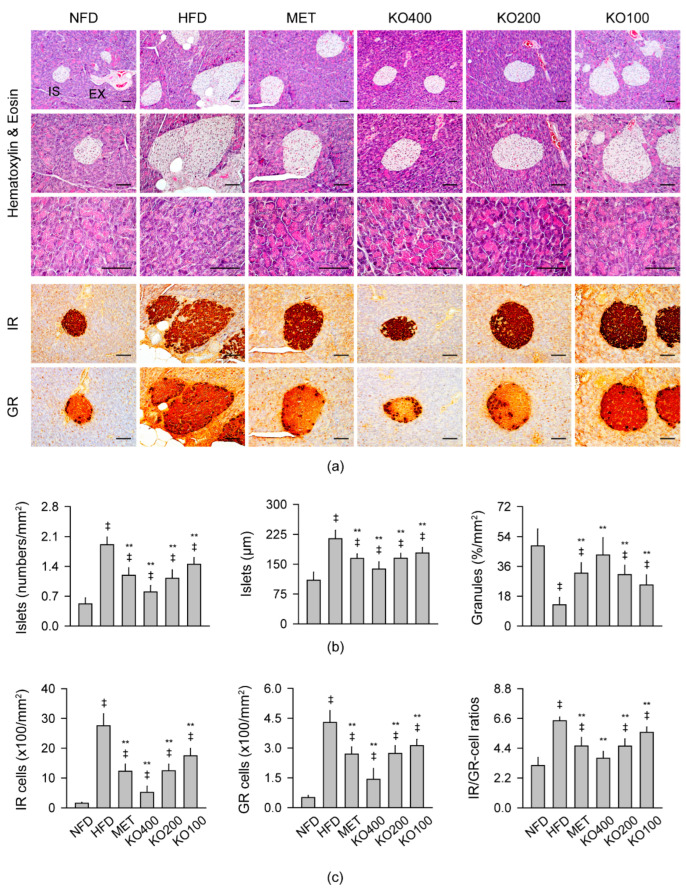
Histopathological changes in the pancreas: (**a**) Representative images in stains with hematoxylin and eosin (HE) and immunostains for insulin (IR) and glucagon (GR). In the HE stains, pancreatic islet (IS) and the exocrine duct (EX) of the acinar region (upper) were high-magnified in the middle and lower area, respectively. Scale bars = 50 μm. (**b**) The number and size of the islets and acinar region containing zymogen granules. (**c**) The number of IR and GR cells and the ratio of IR to GR cells. Values are expressed as the mean ± SD. ^‡^ *p* < 0.01 versus the NFD; ** *p* < 0.01 versus the HFD.

The HFD control exhibited increased and enlarged pancreatic islet with reduced exocrine zymogen granules in HE stains and increased immunoreactive cells for insulin (IR) and glucagon (GR) in the immunohistochemistry (Figure 6). However, the histopathological changes were attenuated in the treatment groups. There were significant differences in the numbers and sizes of the pancreatic islets and the acinar regions containing zymogen granules among the groups (*p* < 0.01). The islet number and size were increased in the HFD control compared with the NFD (*p* < 0.01); however, they were significantly reduced in the treatment groups compared with the HFD control (*p* < 0.01). Conversely, the regions containing zymogen granules were reduced in the HFD control compared with the NFD (*p* < 0.01); however, they were increased in treatment groups compared with the HFD control (*p* < 0.05). There were also significant differences in the IR and GR cells and the ratios of IR to GR cells among the groups (*p* < 0.01). The immunoreactive cells and the ratios increased in the HFD control compared with the NFD (*p* < 0.01); however, they were significantly reduced in the treatment groups compared with the HFD control (*p* < 0.01). The contents of zymogen granules and ratios of IR to GR cells were similar between the NFD and KO400 groups.

#### 2.8.3. Histopathological Improvements in Kidney and Fat Tissues

**Figure 7:**
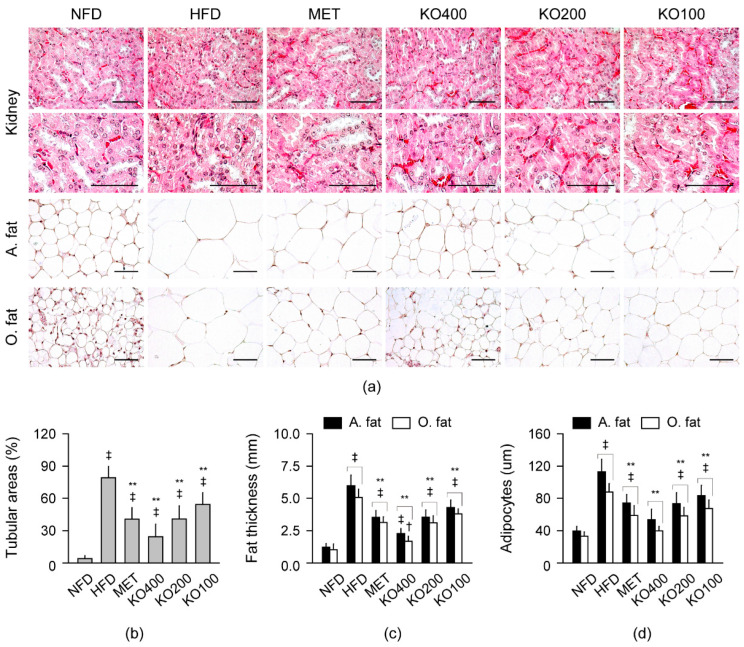
Histopathological changes in the kidney and fat tissue: (**a**) Representative images of the kidney and abdominal (A.) and periovarian (O.) fat tissue in stains with hematoxylin and eosin. Each image was high-magnified in the lower areas. Scale bars = 50 μm. (**b**) Renal tubular area with vacuolation. (**c**,**d**) Thickness of the A. fat and O. fat tissues and the size of their adipocytes. Values are expressed as the mean ± SD. ^‡^ *p* < 0.01 and ^†^ *p* < 0.05 versus the NFD. ** *p* < 0.01 versus the HFD.

In the HE stains, the renal tubular vacuolation and the fat tissue hypertrophy were evident in the HFD control; however, they were mild in the treatment groups (Figure 7). The renal tubular vacuolation, abdominal/periovarian fat tissue thickness, and their adipocyte sizes were significantly different among the groups (*p* < 0.01). The changes significantly increased in the HFD control compared with the NFD (*p* < 0.01); however, they were reduced in the treatment groups compared with the HFD control (*p* < 0.01). The adipocyte sizes in both fat tissues were similar between the NFD and KO400 groups.

## 3. Discussion

The oral administration of KO at three doses of 400, 200, and 100 mg/kg for 12 weeks reduced the HFD-induced weight gains and body fat deposition, in particular in the abdominal region, representing the potential anti-obesity effects. The effects of KO may be involved in promotion of fecal lipid excretion rather than inhibition of HFD intake in energy metabolism. The MET group also showed anti-obese effects along with promoting lipid excretion rather than reduced calories intake, which is similar to previous results [46] but inconsistent with its mechanism regarding inhibition of food intake [11]. The HFD control showed increased blood levels of parameters for NAFLD, T2D, dyslipidemia, and renal dysfunction, which is frequently observed in obese subjects [47,48,49]. Along with these, the HFD-induced metabolic alteration increased the absolute or relative organ weights of the liver, fat tissues, and kidney and reduced the relative weights of the pancreas. However, the changes in the blood parameters and organ weights were significantly reversed by the treatments of KO. Similarly, previous studies have shown inhibitory effects on weight gains and fat accumulation in HFD animal models by oral gavage protocols of KO at 100 to 600 mg/kg for 12 weeks [41,42]. The oral administration also ameliorates NAFLD and dyslipidemia with lower liver damage and adiposity indices, together with a reduction in the blood levels of triglycerides and total/LDL cholesterols and downregulation of hepatic genes involving lipid synthesis (e.g., SREBP1c, FAS, and PPARα) [40,41,42]. Furthermore, many animal studies on the dietary effects of KO supplemented in HFD have shown similar beneficial effects on hepatic steatosis and dyslipidemia as well as improvements in insulin resistance (reviewed in [24,27,28,29,30,31,32,33,34,35,36]). Thus, these results demonstrated convincing inhibitory effects of KO on the progress of obesity and the metabolic syndromes.

The current histopathological analyses support the therapeutic potentials of KO for metabolic syndromes in multiorgans. The elevation of serum triglyceride and total/LDL cholesterol and a decline in HDL cholesterol were accompanied by the hepatocellular and adipocyte hypertrophy and the renal tubular vacuolation in the HFD control; however, the changes were attenuated by the treatments of KO, especially at the higher doses. The lower serum ALP, ALT, AST, GGT, LDH, BUN, and creatinine could be considered as the hepatic and renal protective effects of KO, and the lower glucose, insulin, and HbA1c could be involved in improvements in insulin resistance, similar with previous results in dietary KO [29,30,32,35]. Indeed, KO inhibited pancreatic islet hyperplasia with increases in the β-like (IR) and α-like (GR) cells and the ratios of β- to α-like cells. It is likely that the KO may improve the endocrine function of the pancreas by suppressing the altered insulin secretion for the elevated levels of glucose under insulin resistance. Although glucose/insulin tolerances were not examined here, previous animal studies have reported evidence that KO inhibits insulin resistance and diabetic dyslipidemia by reducing fasting blood glucose and glucose tolerance [34,42]. Furthermore, KO also improved the exocrine function of the pancreas. The HFD control showed atrophic changes of the pancreatic acinar cells and reduced zymogen granules releasing digestive enzymes (e.g., lipase and amylase), similar to previous studies [46,50]. However, KO treatments increased exocrine zymogen granules, which could promote lipid digestion and then fecal lipid excretion [50]. Further mechanism studies are needed to elucidate the functional improvements in the multiorgans.

Obesity leads to a chronic low-grade inflammation in the adipose tissue and the lipid metabolic alteration: chronic FA exposure induces dysfunction of the adipose tissue with stimulation of lipolysis and release of free FAs, resulting in lipotoxicity-induced insulin resistance. The exaggerated availability of free FAs then impairs pancreatic insulin releases and hepatic glucose sensitivities [48,51]. Given that altered lipid metabolism is causally linked to insulin resistance and the related metabolic syndromes, interventions targeting of the dysfunctional adipose tissue can be useful for treating the overall metabolic profile. However, there have been few reports to support the effects of KO on lipid metabolism in adipose tissue. Herein, the HFD-induced adipocyte hypertrophy may result from the excessive lipid storage within the adipocyte and the metabolic alteration for regulating lipid and cholesterol synthesis [47]. The KO downregulated lipogenesis-related genes (i.e., C/EBP-α/-β, SREBP1, PPARγ, and FAS) and upregulated genes related to lipolysis and β-oxidation (i.e., PPARα and UCP2) [52]. The gene-modulating effects of KO may contribute to improvements in endocrine alteration in adipose tissues by downregulating leptin and upregulating adiponectin. Indeed, endocrine dysfunction of the adipose tissue develops insulin resistance as the primary pathogenesis of metabolic syndromes: levels of leptin and adiponectin show positive and negative correlations, respectively, with obesity and severity of NAFLD [53,54]. Leptin stimulates free FA oxidation and glucose uptake, while adiponectin has insulin-sensitizing and anti-inflammatory properties [53]. It is reported that n-3 PUFAs enhance mitochondrial activities in the white adipose tissues and skeletal muscle, and the EPA inhibits insulin resistance and adipose tissue inflammation by increasing adiponectin and reducing pro-inflammatory cytokines [15]. It suggests that lipid synthesis-related gene modification of the KO exerts functional improvements in adipose tissues as the important therapeutic target for obese NAFLD and diabetic dyslipidemia [4].

AMPK signaling regulates lipid and glucose metabolism, and the representative AMPK activator, metformin, is known to have glucose lowering effects by inhibiting hepatic gluconeogenesis and insulin resistance [10]. In previous studies using HFD animal models, dietary KO ameliorates the hepatic steatosis and dyslipidemia through AMPK activation [36], and the oral gavage improves metabolic alterations in the liver through a modification of lipid synthesis-related gene expressions (i.e., PPARα, SREBP1c, and FAS) [42]. In addition, KO has shown anti-inflammatory properties through an inhibition of mitogen-activated protein kinase and c-Jun N-terminal kinase pathways [55]. The current results showed that the KO had glucose lowering effects by stimulating activities of GK for glucose utilization and inhibiting activities of G6pase and PEPCK for the gluconeogenesis. These actions may negatively influence the lipid synthesis by downregulating ACC1 and upregulating AMPK, involved in stimulation of FA oxidation and inhibition of gluconeogenesis and lipogenesis [56,57]. Furthermore, the KO reduced lipid peroxidation (i.e., MDA) and increased antioxidant activities (i.e., GSH, catalase, and SOD) in agreement with previous results in HFD animal model [34,58], and the bioactive antioxidant astaxanthin may contribute to the significant effects [59]. However, there have been inconsistent results showing nonsignificant antioxidant effects in animals [60,61] and clinical studies [62]. The antioxidant mechanism is complicated in obesity-induced metabolic alterations: overflow of free FAs saturates the hepatic mitochondrial β-oxidation and then leads to oxidative stress and inflammation, which impairs T2D [1]. The induced hyperglycemia further activates cellular oxidative stress and inflammation in the pancreas, and gradually deteriorates β-cell functions [51]. In this context, the antioxidant effects of KO may be enhanced, probably by inhibiting the glucotoxicity-/lipotoxicity-induced oxidative stress in addition to its own antioxidant activities [51,63].

In clinical studies, weight changes were not evident in obese subjects receiving daily supplementation of KO at 2 g for 4 weeks or 4 g for 24 weeks [44,64,65]; however, ratios of waist to hip circumferences and visceral adipose tissue to muscular mass were reduced only in the latter dosing [44]. Consistent with the previous animal studies, lower plasma triglycerides were observed in obese subjects by treating KO at 4 g per day for 24 weeks [44], and lower serum triglycerides, cholesterol, and glucose were observed in patients with hyperlipidemia by treating KO at 2–3 g (but not at 1.0–1.5 g) per day for 12 weeks [43]. It is likely that the anti-obesity and antidyslipidemia effects of KO may need the longer treatments at the higher doses, at least more than 2 g. Here, the overall favorable effects of KO were more evident at the higher dose of 400 mg/kg, referring to almost 2 g (1944 mg) in an adult weighing 60 kg as per the following formula: 400 mg/kg (KO dose) × 60 kg (adult weight) × 0.081 (a constant value for body surface area to human). This study had several limitations: the EPA/DHA levels in the KO were not measured, thereby the detailed mechanism of the bioactive components was unclear. In addition, the results in the HFD-induced model could not cover all of the phenomena in genetic obesity and metabolic syndromes. However, this study was the first to demonstrate that treatment of KO via an oral gavage ameliorates HFD-induced obesity and metabolic syndromes including NAFLD, T2D, dyslipidemia, and renal steatosis. Further clinical investigation is needed to address improvements in metabolic alterations in the multiorgans after an oral dose of KO. Antarctic krill is a zooplankton at the bottom of the food chain and the most abundant animal biomass on Earth, with catch limits at less than 10% of the estimated biomass [66,67]. The annual catch of krill is increasing, but the ecological impact seems to be influenced by increased water temperature rather than the large use [68]. Although the cost is a little more expensive for KO than fish oil as a common source of n-3 PUFAs, the high bioavailability of EPA/DHA in KO may provide the cost effectiveness. KO sometimes has side effects including loss of appetites, diarrhea, and constipation; however, the safety profile is found to be Generally Recognized as Safe (GRAS) by the American Food and Drug Administration and obtained a Novel Food status from the European Union [69]. Considering that there is accumulated evidence that a lower ratio of n-6/n-3 PUFAs is a determinant for inhibiting the progress of metabolic syndromes, nutritional supplementation of KO may be a good alternative source for supply of long-chain n-3 PUFAs [15].

## 4. Materials and Methods

### 4.1. Preparation of KO

The commercial product of Antarctic KO (Superba^TM^ Boost) was produced from Aker Biomarine (Houston, TX, USA) and purchased from SC Science (Ilsan, Korea). For contents of phospholipid in KO, KO was diluted in solution of n-hexane and isopropanol (8:2, *v*/*v*), and quantification of phospholipid was conducted by a high-performance liquid chromatography (HPLC) system equipped with Lichrospher 100 Diol column (Merck KGaA, Darmstadt, Germany) at 55 °C. The mobile phases of A and B were n-hexane:isopropanol:acetate:triethylamine at 81.42:17:1.5:0.08 (*v*/*v*/*v*/*v*) and 84.42:14:1.5:0.08 (*v*/*v*/*v*/*v*), respectively. The gradient was composed as follows: 0–5 min, 5–20% B; 5–8.5 min, 20–40% B; 8.5–15 min, 40–100% B; 15–17.5 min, 100–5% B; 17.5–34 min, 5% B. Samples were injected in a volume of 20 μL, and the quantitative data were obtained in a flow rate of 1.0 mL/min for 34 min. The content of phospholipids was calculated under the standard curves. The KO contained 51.2% (*wt*/*wt*) phospholipids including 44.9% phosphatidylcholine, 3.6% 1-palmitoyl-2-hydroxyl-glycero-3-phosphocholine, 2.1% phosphatidylethanolamine, and 0.6% N-Acyl-phosphatidylethanolamine (Figure S1). KO was dissolved in DW and stored at −20 °C until use.

### 4.2. HFD-Induced Mice Model and Treatments

All experimental procedures were approved by the Institutional Animal Care and Use Committee at Daegu Haany University (Gyeongsan, Korea, Approval No. DHU2021-004). Six-week-old female Crlj:CD1 (ICR) mice were obtained from Orient Bio Inc. (Seongnam, Korea). They were housed using a controlled temperature (20–25 °C) and humidity (40–45%) with a 12:12 h light–dark cycle. Standard rodent chow (#38057, Purina feed, Seongnam, Korea) and water were supplied ad libitum. After a week of acclimatization, mice (n = 10 per group) were divided by one NFD group with standard rodent chow (4.0 kcal/g energy with 16% kcal fat) and five HFD model groups with rodent diet with 45 kcal% (4.7 kcal/g energy with 45% kcal fat; #D12451, Research Diet, New Brunswick, NJ, USA). The HFD model mice were adapted to HFD for a week and then regrouped based on similar body weight. The mice received an oral gavage in a volume of 10 mL/kg once a day for 12 weeks as follows: the NFD and HFD control with DW; MET with metformin hydrochloride (Wako, Osaka, Japan) at 250 mg/kg; three KO groups of KO400, KO200, and KO 100 with KO at 400, 200, and 100 mg/kg, respectively. The doses of metformin were determined as described previously [46], and the highest dose of KO referred to that of animal and clinical studies [42,43]. All animals were fasted overnight prior to the initial and last treatments, and body weight was measured daily. After all treatments, mice were euthanized using a CO_2_ gas. The blood was collected, and the liver, pancreas, abdominal/left periovarian fat pads, and left kidney were sampled. The samples were weighed and subjected to the biochemical or histopathological analyses.

### 4.3. Assessment of Daily Energy Intake, Lipid Excretion, and Body Fat Deposition

After supplying diets of 150 g to cages (5 mice in each cage), the consumption for a day was measured every week. The total and fat energies were calculated by multiplying the weight of the consumed diets by their calories. Fecal samples were collected at 8 h after the last treatments, and the lipid contents were assessed using triglyceride colorimetric assay kit (#10010303, Cayman, Ann Arbor, MI, USA) and total cholesterol assay kit (Colorimetric) (#STA-384, Cell Biolabs, San Diego, CA, USA), according to the manufacturer’s protocol. Total body and abdominal fat deposition was assessed using a live DEXA (Medikors, Seongnam, Korea).

### 4.4. Blood Biochemistry

A small volume of whole blood was analyzed for the blood HbA1c using an EasyA1c (Infopia Co., Anyang, Korea). The other samples were collected in sodium fluoride glucose vacuum tube (Becton Dickinson, Franklin Lakes, NJ, USA) for blood glucose level and clotting-activated serum tubes for the serum levels of ALP, ALT, AST, LDH, GGT, BUN, creatinine, triglyceride, total cholesterol, and LDL/HDL. The levels were assessed using a Dri-Chem NX500i (Fuji Medical System Co., Ltd., Tokyo, Japan). Insulin levels were measured using a mouse insulin ELISA kit (#80-INSMS-E01, Alpco Diagnostics, Windham, NH, USA).

### 4.5. Hepatic Antioxidant Activities

Liver tissue of 0.3 g was homogenized in ice-cold 0.01 M Tris-HCl (pH 7.4) for assessing the antioxidant defense system as described previously [46]. Briefly, the level of MDA was assessed using a thiobarbituric acid assay at an absorbance of 525 nm. Contents of GSH was measured at 412 nm using 2-nitrobenzoic acid. Catalase activity was measured at 240 nm as an amount of catalase required to decompose 1 nM of H_2_O_2_ (pH 7.8) per min at 25 °C. SOD activity was estimated as the generation of superoxide radicals produced by xanthine and xanthine oxidase, which react with nitrotetrazolium blue to form formazan dye, and measured at 560 nm. One unit of SOD enzymatic activity was the amount diminishing the initial absorbance of nitroblue tetrazolium by 50% for 1 min. The levels were measured using a plate reader (Tecan, Männedorf, Switzerland), and normalized by the tissue protein contents.

### 4.6. Hepatic Glucose-Regulating Enzyme Activities

Glucose-regulating enzyme activities were examined in 10% homogenates of liver tissue (0.3 g) in 0.1 M triethanolamine, 0.2 M EDTA and 2 mM dithiothreitol as described previously [46]. The homogenates were centrifuged twice at 1000× *g* and at 10,000× *g* for 15 min at 4 °C, and the supernatant was collected. For GK, the supernatant was incubated with the reaction buffer containing 50 mM Hepes-NaGT (pH 7.4), 100 mM KCl, 7.5 mM MgCl_2_, 2.5 mM dithioerythritol, 10 mg/mL albumin, 10 mM glucose, and 4 units glucose-6-phosphate dehydrogenase for 10 min at 37 °C. The activity was measured at 340 nm after incubating with 5 mM ATP at 37 °C for 10 min. For G6pase, the supernatant was measured at 340 nm after incubating with the buffer containing 131.58 mM Hepes-NaGT (pH 6.5), 18 mM EDTA (pH 6.5), 265 mM glucose-6-phosphate, 0.2 M NADP^+^, 0.6 IU/mL mutarotase, and 0.6 IU/mL glucose dehydrogenase for 4 min at 37 °C. For PEPCK, the supernatant was mixed with the buffer containing 72.92 mM sodium Hepes (pH 7.0), 10 mM dithiothreitol, 500 mM NaHCO_3_, 10 mM MnCl_2_, 25 mM NADH, 100 mM inositol-1,4-diphosphate, 200 mM phosphoenolpyruvate, and 7.2 units malic dehydrogenase. The activity was measured at 340 nm as the reduced absorbance using a UV/Vis spectrophotometer (OPTIZEN POP, Mecasys, Daejeon, Korea). All reagents were purchased from Sigma-Aldrich (St. Louise, MO, USA).

### 4.7. Real-Time Reverse Transcription Polymerase Chain Reaction (RT-PCR) Analysis

Gene expressions were assessed as described previously [46]. Total RNA (5 μg) was extracted from the liver and periovarian fat tissue using Trizol reagent (Invitrogen, Carlsbad, CA, USA), and reverse transcribed into complementary DNA (cDNA) using the reagent High-Capacity cDNA Reverse Transcription Kit (Applied Biosystems, Foster City, CA, USA). The contaminating DNA was removed using a recombinant DNase I (Ambion, Austin, TX, USA). The RT-PCR was performed by CFX96^TM^ Real-Time System (Bio-Rad, Hercules, CA, USA), and the PCR condition was as follows: 10 min at 94 °C and 39 cycles of 15 s at 94 °C, 20 s at 57 °C and 30 s at 72 °C. The expression was analyzed by the ABI Step One Plus Sequence Detection System (Applied Biosystems) and normalized to that of GAPDH as an internal control by the comparative threshold cycle method [70]. The primer sequences for the RT-PCR are listed in Table S1.

### 4.8. Histopathology

Tissue samples of the liver, pancreas, fat tissue, and kidney were fixed in 10% neutral buffered formalin. They were then paraffin-embedded and serially sectioned at a thickness of 3–4 μm. The sections were stained with HE, and the other sections of the pancreas were immunostained for insulin and glucagon, as described previously [46]. A portion of the liver was dehydrated in 30% sucrose solutions, and frozen-sectioned at 20 μm, followed by staining in Oil red O. The histomorphometric analyses were examined as follows: diameter of the hepatocytes and adipocytes in 10 cells at least, thickness of fat tissues, pancreatic acinar area occupying zymogen granules, number and diameter of the pancreatic islets, number of vacuolated renal tubules, and Oil red O-stained region. The analyses were performed using a computer-assisted automated image analyzer (*i*Solution FL ver 9.1, IMT *i*-solution Inc., Vancouver, BC, Canada) by a histopathologist blinded to the groups.

### 4.9. Immunohistochemistry

The other sections of the pancreas were deparaffinized and rehydrated, followed by antigen retrieval pretreatment in 10 mM citrate buffer (pH 6.0) for 20 min at 95–100 °C. The endogenous peroxidase was inactivated by 3% H_2_O_2_ in methanol for 30 min. After blocking with horse serum blocking solution (Vector Lab., Burlingame, CA, USA; dilution of 1:100) for 1 h, the sections were incubated with guinea pig anti-insulin (#ab7842; Abcam, Cambridge, UK; dilution of 1:100) and rabbit antiglucagon antibodies (#ab133195; Abcam; dilution of 1:100) at 4 °C overnight. Then, following incubation with biotinylated secondary antibody (Vector Lab.; dilution of 1:50) for 1 h and ABC reagents (#PK-6200; Vector Lab.; dilution of 1:50) for 30 min, the immunoreactivity was visualized with a peroxidase substrate kit (#SK-4100; Vector Lab.; dilution of 1:50) for 3 min and counterstained with hematoxylin. The staining was conducted in a humidity chamber and rinsed in 0.01 M phosphate-buffered saline three times between each step. The cells occupying more than 20% of immunoreactivities were counted.

### 4.10. Statistical Analyses

Data are presented as the mean ± standard deviations of 10 sample sizes. Normal distribution of the variables and homogeneity of variance were examined by the Kolmogorov–Smirnov and Levene tests, respectively. Because of a normal distribution of the variables, data were examined by one-way ANOVA. Kinetic body weight changes were examined by two-way ANOVA to determine the main factors for the groups and the weeks or their interactions. The main factor of the weeks was treated as a repeated measure. The multiple comparison was examined by Tukey HSD and Dunnett’s T3 post hoc tests in cases of equal and nonequal variances, respectively. *p*-Values less than 0.05 were considered statistically significant.

## Figures and Tables

**Table 1:** Organ weight changes in HFD-induced metabolic alteration.

	NFD	HFD	MET	KO400	KO200	KO100
Liver	1.29 ± 0.07 (4.3 ± 0.2)	1.90 ± 0.10 ^‡^ (4.5 ± 0.4)	1.63 ± 0.11 ^‡,^** (4.5 ± 0.3)	1.44 ± 0.10 ^‡,^** (4.3 ± 0.4)	1.61 ± 0.10 ^‡,^** (4.6 ± 0.4)	1.71 ± 0.06 ^‡,^** (4.6 ± 0.3)
Pancreas	0.25 ± 0.04 (0.8 ± 0.1)	0.24 ± 0.03 (0.6 ± 0.1 ^‡^)	0.26 ± 0.02 (0.7 ± 0.1 **)	0.26 ± 0.02 (0.8 ± 0.1 **)	0.26 ± 0.01 (0.7 ± 0.0 **)	0.25 ± 0.02 (0.7 ± 0.1 ^‡,^*)
Kidney	0.21 ± 0.01 (0.7 ± 0.0)	0.30 ± 0.01 ^‡^ (0.7 ± 0.1)	0.26 ± 0.01 ^‡,^** (0.7 ± 0.0)	0.24 ± 0.01 ^‡,^** (0.7 ± 0.0)	0.25 ± 0.01 ^‡,^** (0.7 ± 0.0)	0.27 ± 0.01 ^‡,^** (0.7 ± 0.0)
A. fat	0.07 ± 0.03 (0.2 ± 0.1)	0.44 ± 0.08 ^‡^ (1.1 ± 0.2 ^‡^)	0.24 ± 0.05 ^‡,^** (0.7 ± 0.2 ^‡,^**)	0.18 ± 0.05 ^‡,^** (0.5 ± 0.2 ^‡,^**)	0.24 ± 0.05 ^‡,^** (0.7 ± 0.2 ^‡,^**)	0.30 ± 0.03 ^‡,^** (0.8 ± 0.1 ^‡,^**)
O. fat	0.08 ± 0.03 (0.3 ± 0.1)	0.45 ± 0.11 ^‡^ (1.1 ± 0.3 ^‡^)	0.23 ± 0.05 ^‡,^** (0.6 ± 0.1 ^‡,^**)	0.15 ± 0.03 ^‡,^** (0.4 ± 0.1 ^†,^**)	0.22 ± 0.03 ^‡,^** (0.6 ± 0.1 ^‡,^**)	0.27 ± 0.03 ^‡,^** (0.7 ± 0.1 ^‡,^*)

Absolute organ weights (g) of the liver, pancreas, kidney, abdominal (A.), and periovarian (O.) fat masses were measured, and the relative organ weights to the body weight (%) are indicated in parenthesis. Values are expressed as the mean ± standard deviation (SD). ^‡^ *p* < 0.01 and ^†^ *p* < 0.05 versus the NFD. ** *p* < 0.01 and * *p* < 0.05 versus the HFD control (HFD).

**Table 2:** Blood biochemical analyses in HFD-induced metabolic alteration.

Parameters	NFD	HFD	MET	KO400	KO200	KO100
ALT (IU/dL)	4.8 ± 1.1	15.1 ± 1.3 ^‡^	9.8 ± 1.2 ^‡,^**	7.8 ± 1.4 ^‡,^**	9.8 ± 1.4 ^‡,^**	12.0 ± 1.1 ^‡,^**
AST (IU/dL)	8.5 ± 2.0	20.6 ± 2.3 ^‡^	14.2 ± 1.6 ^‡,^**	11.2 ± 1.6 ^†,^**	14.1 ± 1.3 ^‡,^**	16.4 ± 1.4 ^‡,^**
ALP (IU/dL)	7.7 ± 1.4	24.0 ± 5.3 ^‡^	14.0 ± 1.8 ^‡,^**	10.0 ± 1.3 ^†,^**	14.3 ± 1.9 ^‡,^**	16.7 ± 1.3 ^‡,^*
LDH (IU/mL)	0.6 ± 0.2	4.1 ± 0.9 ^‡^	2.2 ± 0.5 ^‡,^**	1.6 ± 0.5 ^†,^**	2.2 ± 0.4 ^‡,^**	2.8 ± 0.3 ^‡,^**
GGT (IU/dL)	0.4 ± 0.2	1.7 ± 0.2 ^‡^	1.1 ± 0.2 ^‡,^**	0.8 ± 0.2 ^‡,^**	1.1 ± 0.2 ^‡,^**	1.3 ± 0.1 ^‡,^**
Glucose (mg/mL)	1.0 ± 0.1	2.4 ± 0.4 ^‡^	1.5 ± 0.2 ^‡,^**	1.2 ± 0.2 **	1.5 ± 0.2 ^‡,^**	1.7 ± 0.3 ^‡,^**
Insulin (ng/mL)	0.6 ± 0.1	2.7 ± 0.6 ^‡^	1.5 ± 0.3 ^‡,^**	1.2 ± 0.2 ^‡,^**	1.6 ± 0.3 ^‡,^**	1.9 ± 0.2 ^‡,^*
HbA1c (%)	2.7 ± 1.0	9.1 ± 1.3 ^‡^	5.2 ± 1.0 ^‡,^**	4.0 ± 1.1 **	5.3 ± 1.0 ^‡,^**	6.3 ± 1.0 ^‡,^**
BUN (mg/mL)	0.3 ± 0.1	1.3 ± 0.2 ^‡^	0.7 ± 0.1 ^‡,^**	0.6 ± 0.2 ^‡,^**	0.7 ± 0.1 ^‡,^**	0.9 ± 0.2 ^‡,^**
CRE (mg/dL)	0.6 ± 0.2	2.1 ± 0.3 ^‡^	1.4 ± 0.2 ^‡,^**	1.0 ± 0.3 ^†,^**	1.4 ± 0.2 ^‡,^**	1.5 ± 0.1 ^‡,^**
TG (mg/mL)	0.8 ± 0.1	2.4 ± 0.2 ^‡^	1.5 ± 0.3 ^‡,^**	1.1 ± 0.1 ^†,^**	1.5 ± 0.2 ^‡,^**	1.8 ± 0.2 ^‡,^**
TChol (mg/mL)	0.8 ± 0.2	2.7 ± 0.5 ^‡^	1.6 ± 0.2 ^‡,^**	1.3 ± 0.3 ^†,^**	1.6 ± 0.2 ^‡,^**	1.9 ± 0.2 ^‡,^**
LDL (mg/mL)	0.2 ± 0.1	0.8 ± 0.1 ^‡^	0.5 ± 0.1 ^‡,^**	0.3 ± 0.1 ^‡,^**	0.5 ± 0.1 ^‡,^**	0.6 ± 0.1 ^‡,^*
HDL (mg/mL)	0.9 ± 0.2	0.2 ± 0.1 ^‡^	0.5 ± 0.1 ^‡,^**	0.7 ± 0.2 **	0.5 ± 0.1 ^‡,^**	0.4 ± 0.1 ^‡,^**

Blood biochemistries were examined for blood levels of glycated hemoglobin (HbA1c) and serum levels of alanine aminotransferase (ALT), aspartate aminotransferase (AST), alkaline phosphatase (ALP), lactate dehydrogenase (LDH), gamma-glutamyltransferase (GGT), glucose, insulin, blood urea nitrogen (BUN), creatinine (CRE), triglyceride (TG), total cholesterol (TChol), and low-/high-density lipoprotein cholesterol (LDL/HDL). Values are expressed as the mean ± SD. ^‡^ *p* < 0.01 and ^†^ *p* < 0.05 versus the NFD. ** *p* < 0.01 and * *p* < 0.05 versus the HFD.

## Data Availability

Data are contained within the article and the Supplementary Materials.

## Supplementary Materials

The following supporting information can be downloaded at: https://www.mdpi.com/article/10.3390/md20080483/s1, Figure S1: HPLC analysis of KO for phospholipid contents; Table S1: Primer sequences for real-time RT-PCR.
