# Supplementary material for: Preventive and Therapeutic Effects of Krill Oil on Obesity and Obesity-Induced Metabolic Syndromes in High-Fat Diet-Fed Mice
Source: Mar Drugs. 2022 Jul 27;20(8):483. doi: 10.3390/md20080483 (PMC9410137; doi:10.3390/md20080483)
Supplement: Supplementary file 1 [file marinedrugs-20-00483-s001.zip › marinedrugs-1807908-supplementary.pdf]

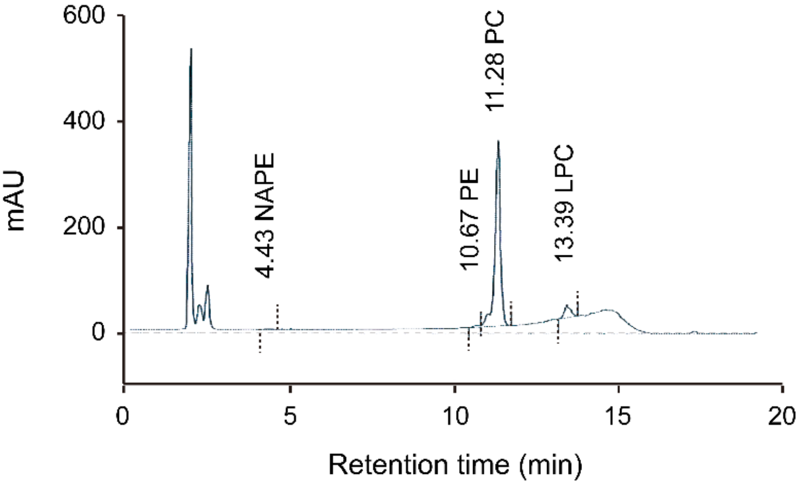

Figure S1. HPLC analysis of KO for phospholipid contents. LPC = 1-palmitoyl-2-hydroxyl-glycero-3-phosphocholine; NAPE = N-Acyl-phosphatidylethanolamine; PC = phosphatidylcholine; PE = phosphatidylethanolamine.

Table S1. Primer sequences for real-time RT-PCR.

Targets (GenBank IDs)	Sequence (5′ – 3′)
ACC1 (NM133360)	*Forward*: GCCATTGGTATTGGGGCTTAC<br>*Reverse*: CCCGACCAAGGACTTTGTTG
Adiponectin (NM009605)	*Forward*: CCCAAGGGAACTTGTGCAGGTTGGATG<br>*Reverse*: GTTGGTATCATGGTAGAGAAGAAAGCC
AMPKα1 (XM011245321)	*Forward*: AAGCCGACCCAATGACATCA<br>*Reverse*: CTTCCTTCGTACACGCAAAT
AMPKα2 (NM178143)	*Forward*: GATGATGAGGTGGTGGA<br>*Reverse*: GCCGAGGACAAAGTGC
C/EBPα (NM001287523)	*Forward*: TGGACAAGAACAGCAACGAGTAC<br>*Reverse*: CGGTCATTGTCACTGGTCAACT
C/EBPβ (NM001287739)	*Forward*: AAGCTGAGCGACGAGTACAAGA<br>*Reverse*: GTCAGCTCCAGCACCTTGTG
FAS (NM007988)	*Forward*: GCTGCGGAAACTTCAGGAAAT<br>*Reverse*: AGAGACGTGTCACTCCTGGACTT
Leptin (NM008493)	*Forward*: CCAAAACCCTCATCAAGACC<br>*Reverse*: GTCCAACTGTTGAAGAATGTCCC
PPARα (NM011144)	*Forward*: ATGCCAGTACTGCCGTTTTC<br>*Reverse*: GGCCTTGACCTTGTTCATGT
PPARγ (NM001127330)	*Forward*: AGTGGAGACCGCCCAGG<br>*Reverse*: GCAGCAGGTTGTCTTGGATGT
UCP2 (NM011671)	*Forward*: CCGCATTGGCCTCTACGACTCT<br>*Reverse*: CCCCGAAGGCAGAAGTGAAGTG
GAPDH (NM008084)	*Forward*: CATCTTCCAGGAGCGAGACC<br>*Reverse*: TCCACCACCCTGTTGCTGTA

ACC1 = acetyl-CoA carboxylase 1; AMPK = 5' adenosine monophosphate-activated protein kinase; C/EBP = CCAAT-enhancer-binding protein; FAS = fatty acid synthase; GAPDH = glyceraldehydes 3-phosphate dehydrogenase; PPAR = peroxisome proliferator-activated receptor; RT-PCR = reverse transcription polymerase chain reaction; SREBP = sterol regulatory element-binding protein; UCP = mitochondrial uncoupling protein.
